# Edge-Termination and Core-Modification Effects of Hexagonal Nanosheet Graphene

**DOI:** 10.3390/molecules19022361

**Published:** 2014-02-21

**Authors:** Jin-Pei Deng, Wen-Hao Chen, Shou-Po Chiu, Chih-Hsun Lin, Bo-Cheng Wang

**Affiliations:** Department of Chemistry, Tamkang University, Tamsui, New Taipei City 25137, Taiwan

**Keywords:** hexagonal grapheme nanosheets, armchair edge, zigzag edge

## Abstract

Optimized geometries and electronic structures of two different hexagonal grapheme nanosheets (HGNSs), with armchair (*n*-A-HGNS, *n* = 3–11) and zigzag (*n*-Z-HGNS, *n* = 1–8) edges have been calculated by using the GGA/PBE method implemented in the SIESTA package, with the DZP basis set, where *n* represents the number of peripheral rings. The computed HOMO-LUMO energy gap (E_g_ = E_LUMO_ − E_HOMO_) decreases for fully H-terminated A- and Z-HGNSs with increasing *n*, *i.e.*, with increasing nanosheet size and *p_π_*-orbitals being widely delocalized over the sheet surface. The full terminations, calculated with various functional groups, including the electron-withdrawing (F-, Cl-, and CN-) and -donating (OH-, and SH-) substitutions, were addressed. Significant lowering of E_HOMO_ and E_LUMO_ was obtained for CN-terminated HGNS as compared to those for H-terminated ones due to the mesomeric effect. The calculated E_g_ value decreases with increasing *n* for all terminations, whereby for the SH-termination in HGNS, the termination effect becomes less significant with increasing *n*. Further, the calculation results for stabilities of HGNS oxides support the tendency toward the oxidative reactivity at the edge site of the sheet, which shows most pronounced C-C bond length alternation, by chemical modification. Physical properties of HGNSs with various numbers of the core-defects, which can be obtained by strong oxidation, were also investigated. Their structures can change drastically from planar to saddle-like shapes. These conformations could be used as stationary phases with controlled interaction in the separation methods such as HPLC and the other chemical analysis techniques.

## 1. Introduction

The recent discovery of grapheme [1], a single atomic sheet of graphite, has ignited intense research activities to elucidate the electronic properties of this novel two-dimensional *p_π_*-conjugated extended system. Graphene is one of the most important subjects in the current materials science study since it has *sp^2^* carbon atoms with very unusual and interesting electronic, thermal, optical, and mechanical properties [2,3,4,5]. Graphene nanoribbons with perfect edges are predicted to exhibit interesting electronic and spintronic properties [6,7,8,9], notably, quantum-confined energy band gaps and magnetic edge states.

Recently, finite-size graphene nanosheets with different shape (e.g., triangular, rectangular, and hexagonal nanosheets) were proposed by theoretical and experimental studies [10,11,12,13,14,15]. The physical and chemical properties of these graphene nanosheets correspond to 2-D quantum dot features due to their size, edged shape and termination which could make them behave as conductors and semiconductors. The effect of electron confinement leads to size-dependent electronic properties in graphene nanostructures. Interestingly, properties of hexagonal graphene nanosheet (HGNS) are also dependent on the shape of its edges, namely, zigzag (Z-HGNS) and armchair (A-HGNS) [16]. The physical properties of HGNS were investigated using the tight binding model, which predicts Z-HGNS and A-HGNS to have metallic and semiconducting properties, respectively [16,17]. Palacios *et al.* explored the magnetic properties of Z- and A-HGNS using theoretical and experimental studies [14]. Enoki and Tan *et al.* proposed the theoretical and experimental cutting graphene into nanographene as well as its edge chlorination [18,19,20]. Using hybrid density functional theory methods, Nakano *et al.* investigated second hyper-polarizabilities of HGNFs. Z-HGNF is also a promising candidate for an open-shell singlet system [21]. The electronic state of HGNF containing defects has been investigated by means of DFT and direct molecular orbital-molecular dynamics methods by Kawabata *et al.* They showed that an excess electron is easily trapped in the defect sites of HGNS making it a stable structure [22]. The hypothesis that HGNS with a size large enough exhibit anti-ferromagnetically ordered and hexagonally sectored spin densities could exist was found to be unjustified by Deleuze *et al.* using spin-free DFT calculations [23]. Karlický *et al.* generated the band structure of graphene halides using DFT with GGA and LDA functionals [24]. Yakobson *et al.* studied the electronic and magnetic properties of hybrids containing graphene and hexagonal boron nitride that occur as phase-separated domains. Quantum dots and nanorods of graphene embedded in hexagonal boron nitride are semiconducting, with a band gap tunable by controlling the size of the nanosheet [25]. Luo *et al.* proposed the mechanism of zigzag edge selectivity and the formation of highly ordered crystallites for producing HGNS over large areas. Their Raman spectra show that the edges of the hexagonal crystallites are predominantly oriented along the zigzag direction [26]. Geng *et al.* proposed a mechanism based on the fast precipitation of carbon atoms and the quick assembly of the growing hexagonal platelets on molten droplets and tried to control the shapes and assemblages of graphene [27].

In the present work, we use the GGA/PBE method with the DZP basis set in the SIESTA package to generate optimized geometries and electronic structures of *n*-Z-HGNS (*n* = 1–8) and *n*-A-HGNS (*n* = 3–11) (17 HGNS in total). Motivated by the recent experimental progress in HGNS, we have carried out DFT calculations to explore the relationship between the calculated E_g_ and the nanosheets’ size [14,19,20]. For all of optimized geometrical structures, related E_g_ values were computed for fully H-terminated A- and Z-HGNS. Because HGNS derivatives are useful in achieving the desired chemical properties suitable for potential applications, chemically modified HGNS with edge-termination at the periphery of graphene were also investigated. Thus, the full terminated A- and Z-HGNS with various substituents, including electron-withdrawing (F-, Cl-, and CN-), and -donating (OH-, and SH-) groups were calculated. At the same time, the regional position of the functional group such as epoxide was further varied to observe its influence on the reactivity. The roles in the chemical reactivity of HGNS of edge and vacancy defects which can be generated by acid treatment and oxidation, were also investigated in the present paper. According to the optimized geometries, the presence of a vacancy defect in HGNS changes its structure from planar (normal HGNS) to saddle- or twisted-like. These calculation results should provide more information on nano-scale phenomena, including structural and electronic properties, for both A-HGNS and Z-HGNS that should be helpful for the development of new technologies with applications ranging from nano-electronics to nano-mechanical devices and chemical analysis techniques.

## 2. Results and Discussion

**Figure 1:**
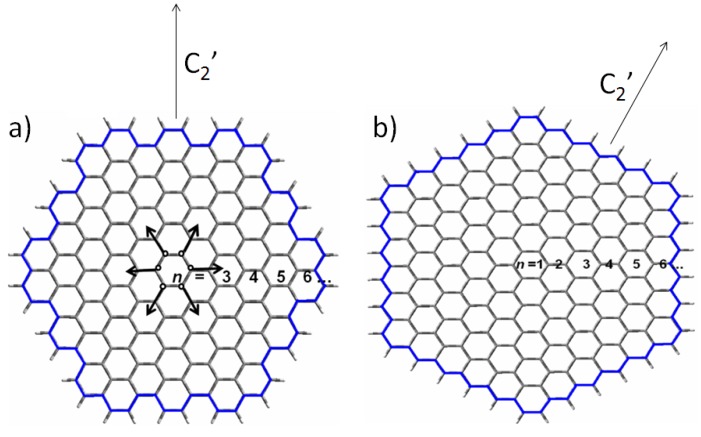
Schematic representation of (**a**) an armchair-graphene (6-A-HGNS) and (**b**) zigzag-graphene (6-Z-HGNS). The white balls denote hydrogen atoms terminating the edge carbon atoms, and the black balls represent carbon atoms. Both A- and Z-HGNS have the *D_6h_* point group.

According to the geometry analysis; the hexagonal carbon ring is the core structure for both Z- and A-HGNS. Fused hexagonal rings expand along the *C_2_* symmetry axis as *C_6_* extension within the *D_6h_* point group (Figure 1); thus; for *n*-A-HGNS; *n*- is the number of peripheral rings around the nanosheet. 3-A-HGNS is the smallest A-HGNS system which contains thirteen fused hexagonal rings.

To count the total number of carbon atoms in *n*-A-HGNS the following equations can be used: 6(*n* − 1)^2^ + 18 (odd *n*) and 6(*n* − 1)^2^ + 30 (even *n*). Thus, 4-A-HGNS, 5-A-HGNS and 6-A-HGNS contain 84, 114 and 180 C atoms, respectively. Similarly, *n*-Z-HGNS expands along the C_2_' axis within *D_6h_* symmetry as zigzag circles around the nanosheet. The total number of C atoms in *n*-Z-HGNS is *6n^2^* where *n* is the peripheral zigzag circle of Z-HGNS (e.g., 8-, 9- and 10-Z-HGNS contain 384, 486 and 600 C atoms, respectively). Furthermore, the nanosheet size (the diameter of HGNS) of *n*-A-HGNS and *n*-Z-HGNS can be calculated as follows: D_a_ (*n*-A-HGNS) = (3*n* − 1) × r for odd *n* and (3*n* − 2) × r for even *n*; D_z_ (*n*-Z-HGNS) = [(4*n* − 2) × cos(π/6) × r]; here; r is the C-C bond length assumed to be 1.42 Å.

### 2.1. Size-Dependent Effect

**Figure 2:**
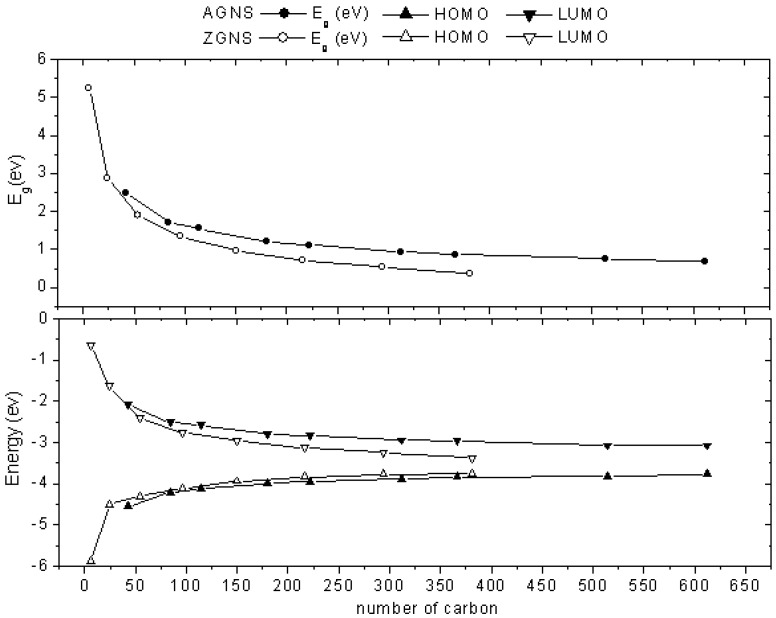
Calculated E_HOMO_, E_LUMO_ and E_g_ values decrease while the size of HGNS increase both for armchair and zigzag types.

According to the solid state physics consideration, an increase of the size of one- or two-dimensional solids may increase the electron conjugation in the system and thus, their calculated E_g_ value decrease. Graphene has C atoms with *sp^2^* hybridization bonding where three neighboring C atoms have an infinite two-dimensional extension forming a honeycomb skeleton with aromatic character. In particular, the free *p_π_* electrons occupying orbitals perpendicular to the graphene plane delocalize in the whole system. Theoretically, these *p_π_* electrons could be free to move in the delocalized system and generate the conducting or semiconducting properties in the surface of graphene. In the present paper, we computed the electronic structures of *n*-A-HGNS (*n* = 3–11) and *n*-Z-HGNS (*n* = 1–8), including E_LUMO_, E_HOMO_ and E_g_ using GGA/PBE methods with the DZP basis set (Figure 2).

The calculated E_g_ value decreases while the size of HGNS increases both for the armchair and zigzag types. Up to the total C atom number over 200 in HGNS, the calculated E_g_ value decreases gradually and reaches the conducting limit. Two sheets of H-terminated HGNS with close sizes (6-Z-HGNS and 7-A-HGNS containing 216 and 234 total C atoms in the nanosheet, respectively) were selected to compare the calculated E_g_ values, 6-Z-HGNS appeared to have E_g_ about 0.4 eV lower than that of 7-A-HGNS (0.71 eV *vs.* 1.12 eV). Hence, Z-HGNS has calculated E_g_ 0.3–0.4 eV lower than that of A-HGNS with the same nanosheet size. According to the previous calculation results, A-HGNS has more terminating H atoms in the edge than Z-HGNS and these terminating H atoms share electrons with nanosheets of the same size. Thus, these calculations expose that Z-HGNS have stronger *p_π_*-conjugation than in A-HGNS. This result is consistent with the Yakobson’s previous calculations [25].

### 2.2. Edge-Termination Effect

**Figure 3:**
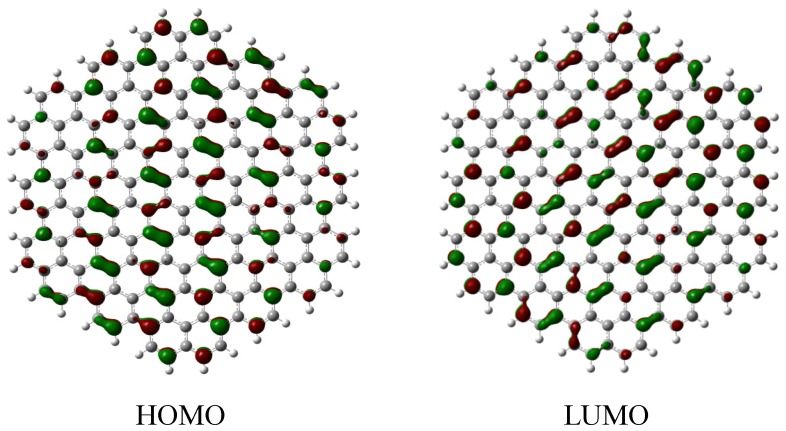
Calculated HOMO and LUMO of H-terminated 6-A-HGNS.

The previous calculation results indicated that the calculated E_g_ value should decrease when the diameter of HGNS increases. In addition, the edge-termination may affect *p_π_*-conjugation for the whole system, thus, the calculated E_HOMO_, E_LUMO_ and E_g_ values change depending on different edge terminators. Theoretically, the edge C atom of Z-HGNS has an unpaired electron and can form a *sp^2^* hybridized bond with some terminating atom or group. The C atom of A-HGNS has a C-C double or single bond between two open edge C atoms, and thus, it may form *sp^2^* and *sp^3^* hybridized bonds with one or two terminating groups, respectively. Chabal *et al.* indicated that the edge C atom of the armchair type is more reactive than that of the zigzag type [28]. For the fully terminated of 6-A-HGNS (Table 1), the electron-withdrawing edge termination results a smaller calculated E_g_ decrease (F-, Cl- and CN- as 1.114 eV, 0.985 eV and 0.972 eV) than that observed for electron-donating terminations (OH- and SH- as 0.873 eV and 0.825 eV) as compared to the H-terminated systems (1.210 eV). Similarly, the F-, Cl- and CN- terminated systems have a smaller decrease in E_g_ (0.655 eV, 0.539 eV and 0.511 eV) than those with OH- and SH- terminations (0.543 eV and 0.411 eV) and as compared the H-terminated 6-Z-HGNS (0.710 eV) (Table 2); these calculated results are consistent with the experimental data [18]. We conclude that fully terminated 6-A-HGNS has the apparent calculated E_g_ value decrease more than that for 6-Z-HGNS and HGNS terminated with electron donating groups exhibit a larger edge termination effect than those with electron-withdrawing terminators. According to the molecular orbital analysis of HGNS, the HOMO and LUMO are π and π^*^ orbitals, respectively (Figure 3), and σ-bonding in the skeleton of graphene affect slightly their electronic structures. Regardless of the electron-donating or withdrawing edge terminators, the calculated E_g_ for the terminated HGNS decreased as compared to the H-terminated HGNS. Theoretically, the mesomeric effect (resonance effect) and the inductive effect in chemistry are the terminator properties of that influence the electronic structure of edge terminated HGNS. The inductive effect is characteristic for two unlike atoms bound by a σ-bond, so it does not play an important role for the calculated E_g_ in the edge-terminated HGNS. Furthermore, the mesomeric effect is caused by the *p_π_*-conjugation between a terminator and a C atom of HGNS. The CN- and SH-terminated A-HGNS exhibit an apparent change in their electronic structure compared to the analogous with those of F-, Cl- and OH-terminated system. The CN group contains a triple bond between the C and N atoms, thus, the orbital of the C atom in the sheet skeleton may easily delocalize to the CN bond. This interaction indeed results in a decrease of the distance between CN and the C atom in the periphery. The calculated E_HOCO_ and E_LUCO_ of CN-terminated HGNS decrease notably compared to the H-terminated HGNS (1.966 eV and 2.204 eV decreasing for E_HOMO_ and E_LUMO_, respectively, as 6-A-HGNS); thus, the mesomeric effect may play a more important role in the electronic structure of terminated HGNS. Similar results are also found in the case of the -C≡C-H substituent. The calculated E_g_ value decreases when the diameter of HGNS increases and a slight edge-termination effect with increasing size of HGNS is also observed (see Supplementary Data). These results elucidated that the calculated E_g_ value decreases notably for the SH- and CN-terminated A-HGNS since they contain 2*p_π_* orbitals, which extend the *p_π_*-conjugation from the core structure of HGNS to the termination site.

**Table 1:** Calculated E_HOMO_, E_LUMO_ and E_g_ for fully terminated 6-A-HGNS.

Terminator	E_HOMO_ (eV)	E_LUMO_ (eV)	E_g_ (eV)
H	−4.000	−2.790	1.210
F	−4.216	−3.102	1.114
Cl	−4.010	−3.025	0.985
CN	−5.966	−4.994	0.972
OH	−3.176	−2.303	0.873
SH	−3.705	−2.880	0.825

**Table 2:** Calculated E_HOMO_, E_LUMO_ and E_g_ for fully terminated 6-Z-HGNS.

Terminator	E_HOMO_ (eV)	E_LUMO_ (eV)	E_g_ (eV)
H	−3.840	−3.130	0.710
F	−3.890	−3.235	0.655
Cl	−3.861	−3.322	0.539
CN	−5.933	−5.422	0.511
OH	−3.021	−2.478	0.543
SH	−3.635	−3.224	0.411

### 2.3. Core Modification Effect (Nanosheet Defect)

**Figure 4:**
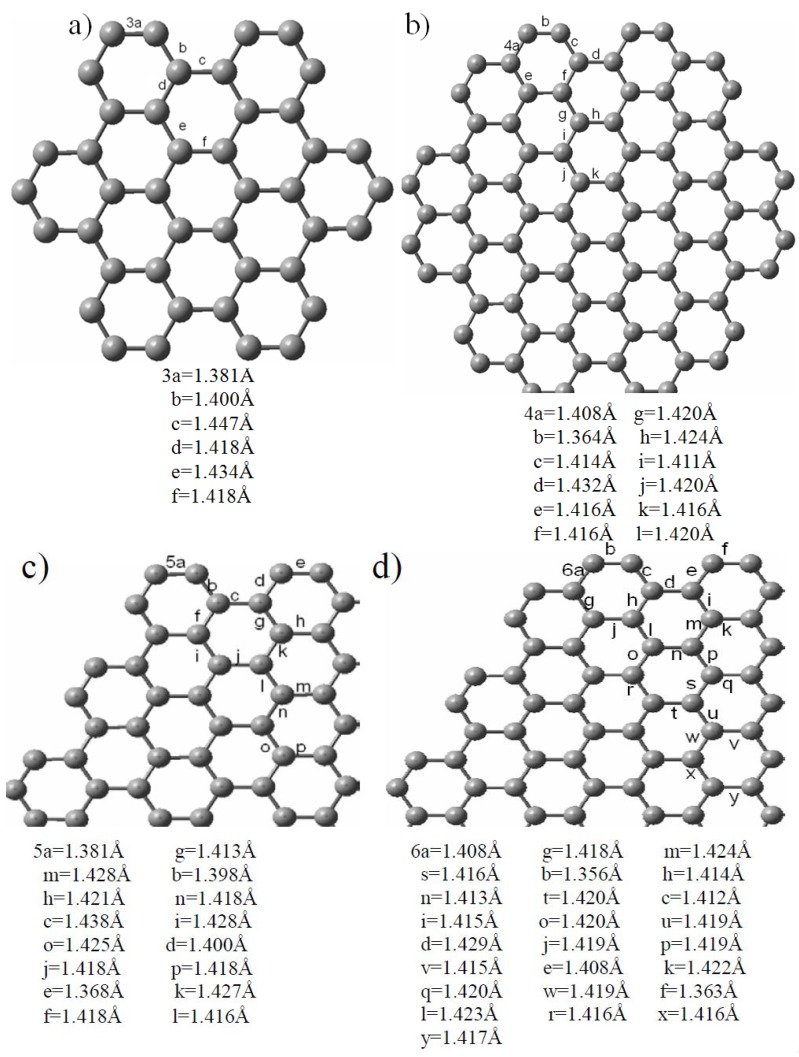
Structures of (**a**) 3-A-HGNS (**b**) 4-A-HGNS (**c**) 5-A-HGNS (**d**) 6-A-HGNS optimized by the PBE/GGA/SIESTA method.

Chemical modification of HGNS core structures is necessary to diversify their reactivity for the demands of fabrication for potential applications. Oxidation, which is usually performed by a strong oxidant or an acid, is a feasible and simple modification method. For example, the reaction of C_60_ and C_70_ in solution with bubbled ozone could form C_60_O and C_70_O (fullerene oxides); thus, similar reactions may occur with the HGNS. The defect formation in the nanoribbon graphene was induced by the acid treatment (HCl), which produced a ribbon edge defect (chemisorption defect) and a ribbon plane defect (vacancy effect). In the present study, two oxide HGNS isomers were proposed to compare the reactivity of HGNS at different reaction sites. First, Figure 4 shows the calculated C-C bond lengths of 3-, 4-, 5- and 6-A-HGNS for comparison; 3a, 4b, 5e and 6b are the shortest bond length in their own sheet. According to the optimized structure of A-HGNS, the calculated length of the bonds located near the sheet edge is shorter than that of the bonds in the core. In particular, the calculated bond lengths are around 1.42 Å and 1.36–1.39Å in the core and the edge, respectively. This result is consistent with Nakano’s calculations [21].

**Figure 5:**
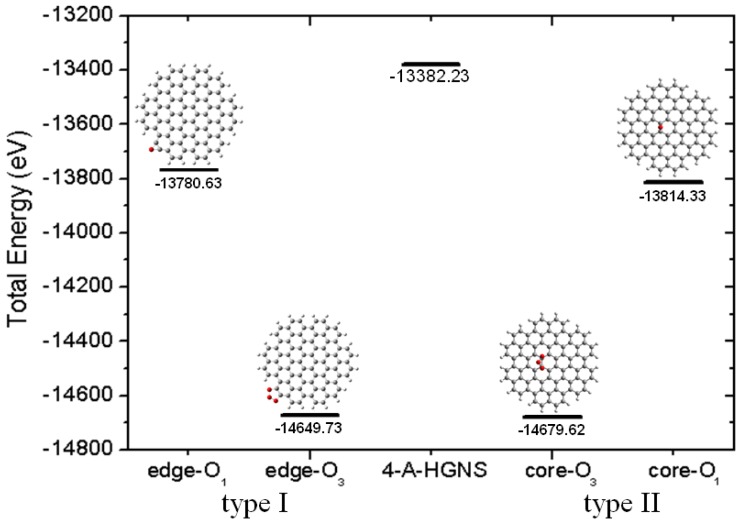
Two isomers of the epoxide substitution 4-A-HGNS considered for comparison of their energies; one has the oxygen atom in the edge (**type I**), and the other has the oxygen atom located in the core of 4-A-HGNS (**type II**). The energies are calculated by the PBE/GGA/SIESTA method.

We conclude that the C-C bonds have a larger double bond character in the sheet corner than in other locations and thus, the C atom in the corner may have a higher reactivity than those in the core. Further, two isomers of the epoxide substitution 4-A-HGNS were considered to compare their energies calculated total energies (Figure 5). One has the oxygen atom on the edge (type I with calculated C-C bond length 1.364 Å) and in the other the oxygen atom is located in the center of 4-A-HGNS (type II with a calculated C-C bond length of 1.420 Å). It is interesting that type I isomer is more stable than the type II one (the calculated total energy difference is 33.7 eV). The results agree with the aforementioned expected reactivity of C=C bonds in HGNS. Because the final products of the oxidation reaction could be controlled by the reaction activation energy rather than by thermal stabilities of the products, two possible intermediate isomers of HGNS epoxides (types I and II) were used to estimate the difference in the activation energy; the proposed structures of the two intermediates (4-A-HGNS-O_3_) originate from the reaction of HGNS with ozone (O_3_). Figure 5 shows that the type I intermediate has the total energy lower (29.89 eV) than that of the type II intermediate, indicating that the type I intermediate should be formed faster via a pathway with lower activation energy and subsequently decomposes to the final product. Furthermore, the two isomers – types I and II, could alter the calculated E_g_ of HGNS: the oxide site close to the core hexagonal ring decreases the calculated E_g_ value. The oxidized position between types I and II of HGNS is closed to that of type II since the calculated C-C bond length consideration. Similarly, 5-A-HGNS has the same calculation results for this isomer investigation. We conclude that the shorter C-C bond length in the corner edge is the active site of HGNS in the oxidation reaction.

**Figure 6:**
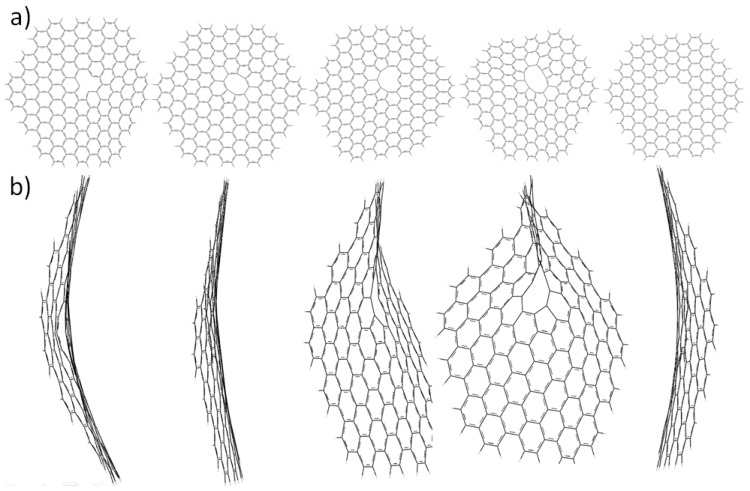
6-A-HGNS containing vacancy defects change their optimized structures from planar to saddle-like: (i) mono- vacancy; (ii) di-vacancy; (iii) tri-vacancy; (iv) tetra-vacancy; and (v) hexa-vacancy; (**a**) elevation view and (**b**) side view.

On the other hand, defect-containing HGNS could be obtained in the production process or by the loss of C atom(s) in the strong oxidation of HGNS oxides via CO or CO_2_ elimination. Several groups have reported theoretical calculations of graphene with defects characterizing its electronic, geometric structures and magnetic properties [29,30,31]. In particular, atomic scale-defects similar to those existing in the nanoribbon graphene could be found in the HGNS. Figure 6a illustrates the vacancy and edge defects in 6-A-HGNS made by removing C atoms from the central hexagonal ring of HGNS in the 1–6 sequence to produce mono, di-, tri-, tetra-, and hexa-vacancy (as plane vacancy defects) as well as two types edge defects in a corner and in an edged. In the present study, a singlet neutral ground state was assumed in the defect-containing HGNS with edge H-termination to generate their electronic and geometric structures by using GGA/SIESTA calculations. Mono-vacancy 6-A-HGNS obtained by removing one C atom from 6-A-HGNS, has three unsaturated satisfied C atoms around the vacancy and the calculated E_g_ value decreases to 0.707 eV. Removal of two nearby C atoms could lead to the formation of one octa-membered ring and two penta-membered rings in an opposite position in di-vacancy 6-A-HGNS, as proposed by Banhart *et al*. [30]. This di-vacancy 6-A-HGNS allows *sp^2^* hybridization for all C atoms and the calculated E_g_ value decreases to 0.523 eV. Tri-vacancy 6-A-HGNS creates two penta- and one deca-membered rings and the calculated E_g_ is 0.231 eV. Tetra-vacancy 6-A-HGNS has the calculated E_g_ value increasing to 1.044 eV since the sheet structure is distorted. Hexa-vacancy 6-A-HGNS obtained by removing one hexagonal ring from 6-A-HGNS has a better *p_π_*-conjugation over the sheet than the other vacancy-defect structures and exhibits the calculated E_g_ of 0.126 eV. Tachikawa *et al.* reported a DFT calculation of the electronic structure of one-vacancy defect 4-Z-HGNSand concluded that the HOMO is widely delocalized over the graphene surface whereas LUMO is localized around the defect site [32]. Qi *et al.* used DFT calculations to consider chemisorptions of oxygen on the defective graphene site [29]. Due to the bond length differences, the vacancy-defective A-HGNS changes its structure from planar to saddle-like (Figure 6b).

The calculated bond length located in the defect site of HGNS is around 1.39 Å, which is close to that of a bond on the edge. For the active site consideration, defect and edge sites have equal possibility to generate the chemisorption of oxygen of HGNS (the calculated HOMO and LUMO of defect HGNS indicated this also). Theoretically, the defect-containing HGNS could be para-(mono- and tri-vacancy 6-A-HGNS as open shell structure) and dia-magnetic (di-, tetra- and hexa-vacancy 6-A-HGNS as closed shell structures).

## 3. Experimental

We have performed the first-principle periodic calculations based on density functional theory adopting the SIESTA package which provides a very useful calculation technique for theoretical studies of periodic systems with a large number of atoms [33,34,35]. The approach uses the standard Kohn-Sham self-consistent density functional method in the local density approximation (LDA) and generalized gradient approximation (GGA) with parameterization of the Perdew-Burke-Ernzerhof (PBE) exchange and correlation functionals with a double zeta polarized (DZP) basis set [36,37,38]. The basis set is a linear combination of numerical atomic orbitals (LCAO), which includes double-zeta polarized orbitals, where (1*s*) for the H valence electron and (2*s*2*p*) for the valence electrons of C atoms were used, and the energy cutoff is 300 Rydberg (the Rydberg constant, 1Ry = hc and R_∞_ = 13.605 eV) to define the finite real-space grid. Using the optimized structures from the GGA/PBE/DPZ/SIESTA calculations, the E_HOMO_, E_LUMO_, and E_g_ (= E_LUMO_ − E_HOMO_) were computed employing the same method.

## 4. Conclusions

The electronic and optimized geometric structures of HGNSs both with armchair and zigzag edges (A- and Z-HGNS) have been generated by using the GGA/PBE method with the DZP basis set implemented in the SIESTA package. HGNSs contain free *p_π_* electrons perpendicular to the planar nanosheet as a 2-D *p_π_*-conjugated extension. Their calculated E_g_ values decrease while the size of the nanosheet increases and approaches a constant. The substituent terminated-HGNS could exhibit lower calculated E_g_ both for A-HGNS and Z-HGNS as compared to the H-terminated systems both with electron-withdrawing (F-, Cl- and CN-) or -donating terminators (OH- and SH-). CN-, OH- and SH-terminated HGNSs display a notable decrease of the calculated E_HOMO_ and E_LUMO_ caused by the mesomeric effect originating from the *p_π_*-conjugation between the core C atom and the terminator. The terminated A-HGNS show a larger decrease of the calculated E_g_ than Z-HGNS with the same terminator. The terminators could tune E_g_ with the planar structure and they could be used in materials for electronic devices. As the interaction with bubbled ozone can produce oxidized HGNS, the oxide position is expected to be in the edge site of sheet based on the C-C bond length consideration. The calculation results for HGNS oxides stabilities support the tendency toward the oxidative reactivity. The nanosheet vacancy defect in HGNS was investigated for mono-, di-, tri-, tetra- and hexa-vacancy; their calculated E_g_ values decrease as compared to the regular 6-A-HGNS except for the hexa-vacancy. The calculated E_g_ value for edge-defect A-HGNS decreases as compared to the regular H-terminated A-HGNS. The optimized structures of vacancy defect-containing HGNS are distorted from the planarity to the saddle-like shape. The calculation results in this work provide a simple theoretical tool to design graphene related materials with desired quantum dot properties.

## Supplementary Materials

Supplementary materials can be accessed at: http://www.mdpi.com/1420-3049/19/2/2361/s1.
