# Supplementary material for: Edge-Termination and Core-Modification Effects of Hexagonal Nanosheet Graphene
Source: Molecules. 2014 Feb 21;19(2):2361–73. doi: 10.3390/molecules19022361 (PMC6271332; doi:10.3390/molecules19022361)
Supplement: Supplementary file 1 [file molecules-19-02361-s001.pdf]

Supplementary Materials

Calculated HOMO and LUMO for original HGNS (AC6 and AC4), defect HGNS (AC4-d6 and AC6-d6), and epoxy-HGNS for core and edge (AC4-expoxy core and edge) which indicated the electron density HGNS.

	HOMO	LUMO
AC6	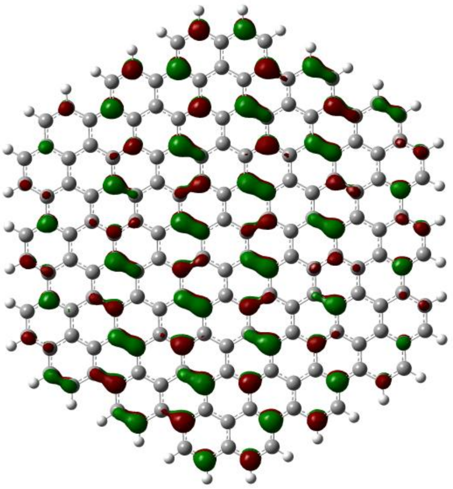	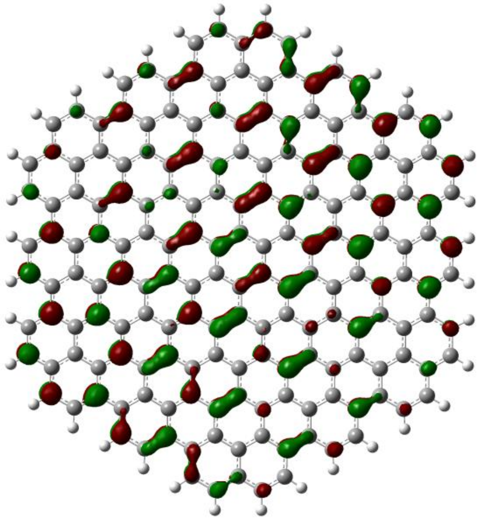
AC6-d6	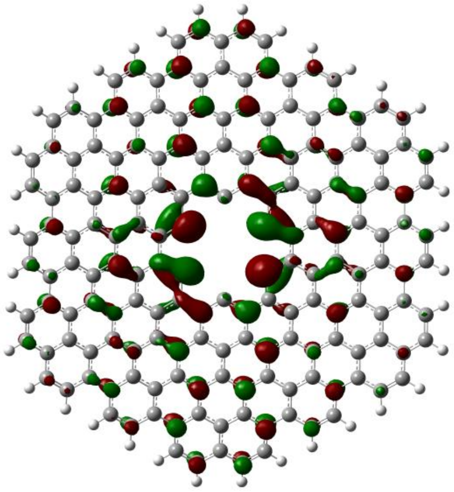	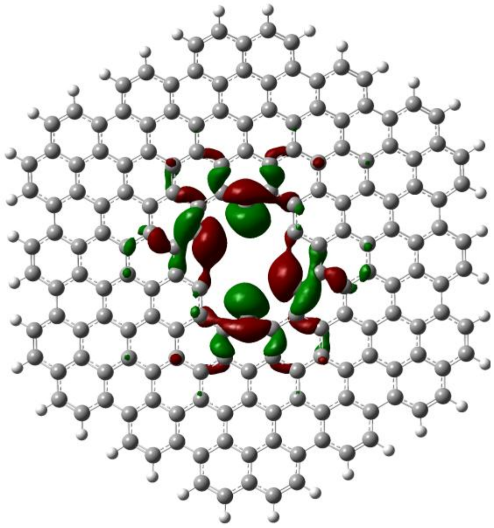
AC4	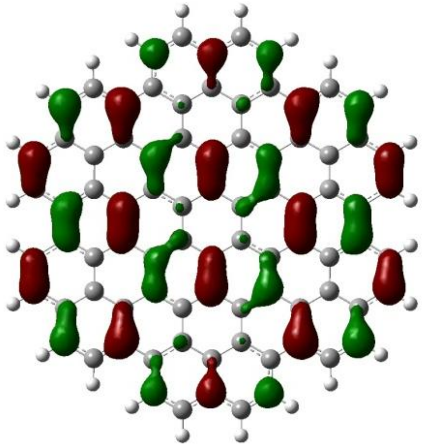	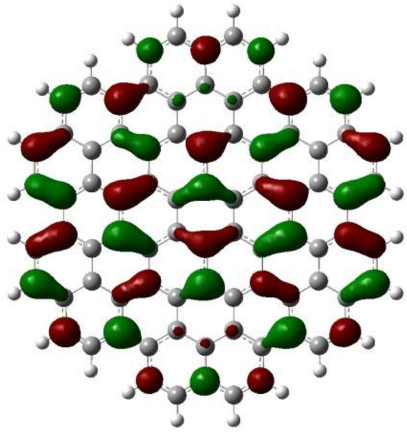

AC4-epoxy-core	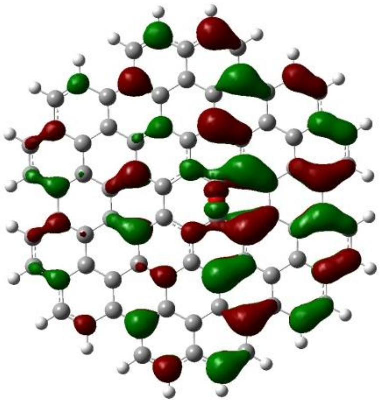	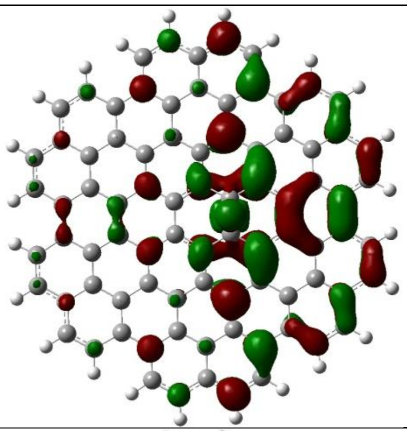
AC4-epoxy-edge	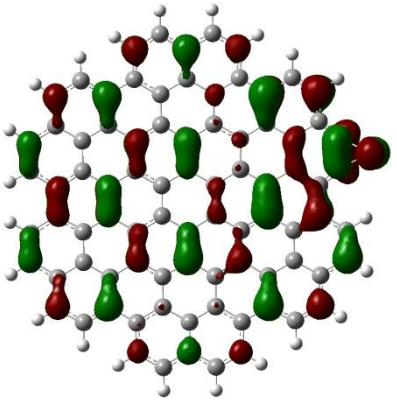	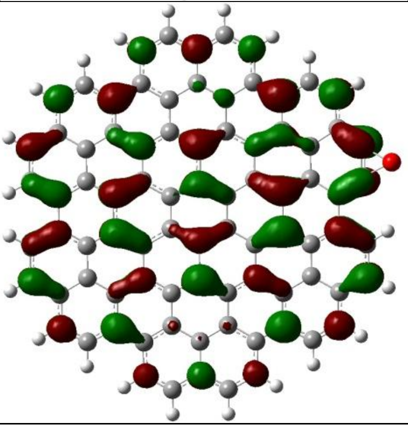
